# A pilot study of biomechanical assessment before and after an integrative training program for adolescents with juvenile fibromyalgia

**DOI:** 10.1186/s12969-016-0103-7

**Published:** 2016-07-22

**Authors:** Susan T. Tran, Staci Thomas, Christopher DiCesare, Megan Pfeiffer, Soumitri Sil, Tracy V. Ting, Sara E. Williams, Gregory D. Myer, Susmita Kashikar-Zuck

**Affiliations:** Department of Psychology, DePaul University, Chicago, IL USA; Division of Sports Medicine, Sports Medicine Biodynamics Center and Human Performance Laboratory, Cincinnati Children’s Hospital Medical Center, Cincinnati, OH USA; Division of Behavioral Medicine and Clinical Psychology– MLC 7039, Cincinnati Children’s Hospital Medical Center, 45229 Cincinnati, OH USA; Department of Pediatrics, Emory University School of Medicine, Children’s Healthcare of Atlanta, Aflac Cancer and Blood Disorders Center, Atlanta, GA USA; Division of Rheumatology, Cincinnati Children’s Hospital Medical Center, Cincinnati, OH USA; Department of Pediatrics, University of Cincinnati, College of Medicine, Cincinnati, OH USA; Department of Orthopaedic Surgery, University of Cincinnati, College of Medicine, Cincinnati, OH USA; The Sports Health and Performance Institute, OSU Sports Medicine, Ohio State University Medical Center, Columbus, OH USA; The Micheli Center for Sports Injury Prevention, Boston, MA USA

**Keywords:** Juvenile fibromyalgia, Chronic pain, Biomechanical assessment, Physical activity, Adolescents

## Abstract

**Background:**

Adolescents with juvenile fibromyalgia (JFM) tend to be very sedentary and avoid participation in physical activity. A prior study suggested that JFM patients show altered biomechanics compared to healthy adolescents which may make them more prone to pain/injury during exercise. A new intervention combining well established cognitive behavioral therapy (CBT) techniques with specialized neuromuscular exercise —Fibromyalgia Integrative Training for Teens (FIT Teens) was developed and shown to be promising in improving functioning in adolescents with JFM. In contrast to traditional exercise programs such as aerobic or resistance training, neuromuscular training is a tailored approach which targets gait, posture, balance and movement mechanics which form the foundation for safe exercise participation with reduced risk for injury or pain (and hence more tolerable by JFM patients). The aim of this pilot feasibility study was to establish whether objective biomechanical assessment including sophisticated 3-D motion analysis would be useful in measuring improvements in strength, balance, gait, and functional performance after participation in the 8-week FIT Teens program.

**Methods:**

Eleven female participants with JFM (ages 12–18 years) completed pre- and post-treatment assessments of biomechanics, including walking gait analysis, lower extremity strength assessment, functional performance, and dynamic postural stability.

**Results:**

Descriptive data indicated that mechanics of walking gait and functional performance appeared to improve after treatment. Hip abduction strength and dynamic postural control also demonstrated improvements bilaterally.

**Conclusions:**

Overall, the results of this pilot study offer initial evidence for the utility of biomechanical assessment to objectively demonstrate observable changes in biomechanical performance after an integrated training intervention for youth with JFM. If replicated in larger controlled studies, findings would suggest that through the FIT Teens intervention, adolescents with JFM can progress towards normalized strength and biomechanics, which may enhance their ability to engage in physical exercise.

**Electronic supplementary material:**

The online version of this article (doi:10.1186/s12969-016-0103-7) contains supplementary material, which is available to authorized users.

## Background

Juvenile fibromyalgia (JFM) is characterized by chronic widespread musculoskeletal pain, occurs in 2–6 % of children, primarily in adolescent girls [1–5], and is associated with significant physical and emotional impairment [6–11]. Typically, symptoms are best managed through a combination of medication, regular exercise, and cognitive-behavioral therapy (CBT) [12–14]. Although routine engagement in physical exercise is strongly recommended for pain management in musculoskeletal conditions such as JFM [12], adherence to exercise in patients with JFM is poor [15, 16] and the majority of individuals with fibromyalgia are sedentary [17–19]. Prolonged sedentary behavior may contribute to physical deconditioning and a loss of confidence in one’s ability to participate in physical activity [20], which reinforces the cycle of pain and disability.

Maintaining adequate daily physical activity is a challenge for patients with JFM because exercise may be difficult or uncomfortable, particularly after a pain flare [21]. Our recent investigation of adolescents with JFM using sophisticated biomechanical analyses documented altered biomechanics, deficits in functional performance, lower strength, and greater fear of movement compared to healthy peers [22]. These altered movement patterns are similar to those that increase risk of injury [23, 24]. It is likely that poor body biomechanics may be a key contributor to patients’ high fear of movement [22] and difficulty adhering to traditional aerobic or resistance training programs. Traditional aerobic or resistance training programs, even those with a graded approach, do not focus on establishing fundamental movement competence prior to implementing the recommended exercises. Evidence from sports medicine and injury prevention research supports the importance of developing core strength, conditioning, and basic movement skills *prior* to engagement in higher levels of physical activity [25, 26].

Tailored exercise programs are needed for fibromyalgia patients [27] to improve their movement competence while simultaneously enhancing psychological confidence in engaging in physical exercise. CBT has been shown to effectively decrease functional disability and depressive symptoms in patients with JFM [28]; however, on its own, CBT did not increase engagement in vigorous physical activity [29]. Integrating behavioral coping skills training, including targeting maladaptive thought patterns such as fear of pain, with a tailored exercise program could have the potential to optimize pain and disability outcomes in JFM.

The Fibromyalgia Integrative Training for Teens (FIT Teens) intervention was created by modifying a specialized integrative neuromuscular exercise program used in injury prevention for young athletes for the needs of adolescents with JFM. FIT Teens incorporates CBT strategies to allow participants to apply pain coping skills in-vivo as they learn the neuromuscular exercises. A qualitative investigation documented the feasibility, tolerability, and initial efficacy of the new FIT Teens program for patients with JFM [30]. Patients reported high levels of engagement, felt they were coping more effectively, experiencing less fatigue, and becoming more active after treatment. Participants’ self-reports on validated measures also reflected improvements in functional disability, depression, fear of movement, pain catastrophizing, and readiness to change [31]. Measurement of objective physical improvements would help to more fully capture the effects of the FIT Teens intervention (beyond self-report) and also better understand the mechanisms of improved physical function. Unfortunately, despite the availability of sophisticated new technologies for assessing gait and functional movements there is very little research in the assessment of movement biomechanics in patients with musculoskeletal pain, and none on adolescents with JFM. The focus of the current study was to examine whether objective biomechanical assessments showed utility and responsiveness to change after the 8-week FIT Teens Program - specifically on measures of gait mechanics, dynamic postural stability, functional performance, and knee and hip strength.

## Methods

### Participants

Youth (12–18 years old) were eligible if they were diagnosed with JFM by a pediatric rheumatologist or pain physician using Yunus and Masi criteria (Table 1) [5]. Additionally, only participants with moderate to high pain-related disability (Functional Disability Inventory score ≥ 12) [32] were included. Participants were excluded if they had a diagnosis of a comorbid rheumatic disease (e.g., juvenile arthritis, systemic lupus erythematous), untreated major psychiatric diagnosis (e.g., major depression, bipolar disorder, and psychosis), documented developmental delay, any medical condition determined by their physician to be a contraindication for exercise, or ongoing participation in CBT for pain. For details, screening and recruitment procedures were described previously [30].

**Table 1:** Yunus and Masi [5] diagnostic criteria for juvenile primary fibromyalgia syndrome

Major Criteria
1. Generalized musculoskeletal pain at three or more sites for three or more months 2. No underlying medical condition 3. Normal laboratory tests 4. Five or more typical tender points
Minor Criteria
Presence of three of the following features: 1. Chronic anxiety or tension 2. Fatigue 3. Poor sleep 4. Chronic headache 5. Irritable bowel syndrome 6. Subjective soft tissue swelling 7. Numbness 8. Pain modulation by physical activities 9. Pain modulation by weather factors 10. Pain modulation by anxiety or stress

Research assistants identified potential participants from pediatric rheumatology and pain clinics at a large children’s hospital. Physicians confirmed medical eligibility and introduced the study to patients and their caregivers. If interested, a research assistant met with families to discuss the study, answer any questions, and obtain written informed consent and assent. This study was approved by the Institutional Review Board of the Children’s Hospital and the rights of participants were protected.

### Biomechanical testing procedures

Prior to and after the intervention, participants completed biomechanical assessments with a trained exercise physiologist at the Children’s Hospital’s Sports Medicine Biodynamics & Human Performance Laboratory. Participants completed measures that assessed walking gait, dynamic postural stability, lower extremity strength, and functional performance that included 3D motion capture technology. During the gait analysis and functional performance assessments, participants wore 43 retroreflective markers placed on standardized locations [22, 33], with a minimum of three markers per segment, including bilateral lower extremities (e.g., foot, shank, and thigh) and trunk (e.g., pelvis and thorax).

#### Gait analysis

Kinematics of walking have been shown to be dependent on gait speed [34], so walking gait was analyzed under two conditions: 1) self-selected pace and 2) standardized pace (1.2 meters/second) at 240 Hz. For the self-selected pace condition, participants were instructed to walk at their normal pace and were given no corrective feedback. During the standardized pace condition, participants were given feedback to adjust their speed (either faster or slower) to standardize their pace by measuring the time it took to walk 3 meters. Normal gait speeds in healthy adults have been shown to range from 1.05 to 1.43 m/s so the standardized pace was set to 1.2 m/s [35, 36]. Participants walked across the laboratory floor which was embedded with force plates (AMTI; Advanced Medical Technology, Inc., Watertown, MA) sampling at 1,200 Hz, and three foot strikes on each side were recorded for each walking condition. Gait was captured using a 10-camera real-time high-speed 3-D motion analysis system (Raptor-E; Motion Analysis Corp., Santa Rosa, CA).

#### Knee strength

Isokinetic knee strength was assessed using the Biodex System II (Shirley, NY). Participants sat in the dynamometer chair secured with a thigh strap and waist strap aligning their hips at 90° and aligning their knees with the rotation axis of the machine. The lower leg was strapped to the dynamometer arm 2 inches above the lateral malleolus. Participants practiced first, performing five continuous repetitions of maximal knee extension/flexion starting at 90° knee flexion; participants extended their knee to full extension and actively flexed the knee back to the starting position at an isokinetic speed of 300°/ second. After a period of rest, participants performed 10 continuous repetitions with maximal effort on each leg. Peak torques (Nm/kg) of knee extension and knee flexion were recorded [37].

#### Hip strength

Hip abduction strength was assessed using the Biodex System III. Participants stood facing the dynamometer head, aligning the center of their hip with the axis of rotation. The testing leg was strapped to the dynamometer arm just above the knee. Participants were instructed to kick out to the side with maximal effort five times and the testing speed was set to 120°/second. Participants practiced first, rested, then performed five repetitions on each leg. Peak torque (Nm/kg) of each kick was recorded [38].

#### Functional performance

To perform the drop vertical jump task (DVJ), participants stood on a 31-cm box with their feet shoulder-width apart. They were instructed to drop off the box with both feet at the same time, land on the force plates in front of them, and immediately jump with both feet to perform a vertical jump with maximal effort towards an overhead target (a basketball). After watching the procedure demonstrated, participants completed practice trials (typically 2–3) until they were comfortable with the test. Participants were provided the opportunity for rest breaks after practicing and between trials; however, most participants did not require breaks. The 3-D motion analysis system was used to assess landing technique during the DVJ [39].

#### Dynamic postural stability

Adolescents with JFM often report difficulty with stability and balance and therefore a standardized tool used in prior studies [40–42] was used to assess postural stability. To perform the Star Excursion Balance Test, participants stood on one lower extremity at the center of the grid. After observing a demonstration of the task, participants were instructed to perform a maximal reach with the non-stance leg in the anterior, posteromedial, and posterolateral direction while maintaining balance on their single leg. The exercise physiologist visually measured the most distal reach distance of the foot in all three directions in centimeters. The process was then repeated while standing on the opposite extremity. Each participant performed at least two complete cycles for practice before the reach distances were recorded. Trials were repeated if the subject was unable to maintain their balance for the duration of the test, if weight was shifted to the reach leg, or if the heel of the stance leg did not maintain contact with the floor.

### Intervention

The FIT Teens intervention was group-based (consisting of 3–4 participants per group) and conducted over eight weeks; participants met twice per week, 90 min per session (~45 min each devoted to CBT and neuromuscular training). Groups met at the Children’s Hospital’s Sports Medicine Biodynamics & Human Performance Laboratory, with the neuromuscular training taking place in a gym setting, and the CBT component in an adjoining conference room. The development and content of the FIT Teens program was fully described in a previous publication [30]. Briefly, the CBT components of the program were based on our published clinical trial [28] and consisted of pain coping skills training including pain education, relaxation skills, distraction, activity pacing, problem solving, and modifying negative and catastrophic thoughts about pain. Coping skills were modified and integrated into the neuromuscular training component in order to demonstrate in-vivo application of coping skills.

The resistive training protocol used in the neuromuscular training was specially designed to reduce incidence of muscle pain and minimize delayed-onset muscle soreness by gradually increasing the complexity of muscle actions. The protocol builds upon the foundations of movement by moving from most basic isometric “hold” exercises, to concentric “muscle shortening” exercises, to eccentric “muscle lengthening” exercises, and finally the full range of motion for “functional movement” (complete protocol published by Thomas et al. [43]). Given known deficits in biomechanics of youth with JFM, the program was tailored to each participant’s baseline levels of ability and confidence in movement, and modified to allow participants to master each level before advancing. The final level is a culmination of all activities, built into functional movements that are relevant for activities of daily life. Additionally, considering that joint hypermobility is a relatively common occurrence in JFM [44], modifications were made as needed to protect joints from hyperextension. At the end of each session, participants were provided home practice instructions for coping skills (practiced every day) and physical exercises. Participants were instructed to practice neuromuscular exercises at home two days over the weekend in addition to the two sessions per week, exercising for a total of four times per week. During each session, homework diaries were reviewed to discuss practice and barriers to practice.

As described by Thomas et al. [43], the exercises included in the intervention were geared towards mimicking activities of daily living. Through each phase of the protocol, specific instructions were given to the participants to encourage proper muscle activation, and therefore optimum technique and body position. For example, the squat exercise was performed at each stage and careful instructions and feedback were given to encourage participants to activate not only their quadriceps, but also the posterior chain muscles (gluteus maximus, gluteus medius, etc.) to obtain and maintain good squat position by ensuring that the knees were not lined up anterior to the toes. In addition, knee alignment in the frontal plane was strongly emphasized such that the knees were not in a valgus position, or came in towards the midline of the body while in the squat position. Focus on knee and hip alignment encouraged activation of the hip abductors and external rotators. Training the muscles to perform in the correct position can assist in a more favorable landing position during functional movements like the DVJ.

### Data analysis

All biomechanical kinematic measures were calculated relative to the participants’ neutral (zero) alignment; measured by the participant standing still aligned with the laboratory coordinate system. All biomechanical data reduction was performed using custom Matlab (Mathworks; Natick, MA) scripts. Kinematic analyses and strength data were inherently standardized for body weight. Basic kinetic data, including gait speed and stride length, were computed.

For the gait analysis, the right and left legs were used to calculate kinematic measures during both walking conditions. The gait cycle was determined, beginning with the heel strike on the force plate and ending with the following heel strike, averaged, and normalized to 101 data points to allow comparisons between the two testing time points (pre- and post-treatment). The heel strike was determined in Visual3D (C-Motion) using a proprietary algorithm (i.e., force > 20 N) [45]. Peak values were determined during the first 60 % of stance, comparable to previous gait studies [46].

Regarding the DVJ, the stance phase, from initial contact with the force plate to the point at which the participant left the force plate, of each trial was determined, averaged, and normalized to 101 data points. Kinematic and kinetic waveforms were used to illustrate the data due to the immense amount of data extracted during the 3-D Motion analysis of the DVJ. The stance phase is depicted within the waveforms for the DVJ.

For dynamic postural control, composite scores were calculated for each participant by dividing the sum of the maximal reach in the anterior (A), posteromedial (PM) and posterolateral (PL) directions by three times the limb length (LL) of the individual, and then multiplying by 100 {[(A + PM + PL)/(LL × 3)] × 100}.

All data were entered and analyzed in SPSS. Descriptive statistics (means and standard deviations) on all biomechanics, strength and postural stability assessments were computed at pre- and post-treatment. Since this was a preliminary study targeted primarily at feasibility and initial evidence of effects, significance testing was not deemed appropriate. Effect sizes (Cohen’s d) for all measures were computed to estimate the potential magnitude of treatment effects.

## Results

Of the 17 initially enrolled JFM participants, 11 participants (*Mean* age = 16.00 years, *SD* = 2.15; 100 % females; 81.8 % Caucasian) completed the FIT Teens intervention. Drop outs were due to schedule conflicts/transportation difficulties (*N* = 4) and need for referral for more intensive psychological/psychiatric care (*N* = 2). All 11 participants completed the dynamic postural stability and strength assessments (dataset available in Additional file 1), 10 completed the walking gait assessment, and eight completed the functional performance assessment (DVJ). One participant was not analyzed for walking gait or DVJ due to equipment difficulties, and two declined the DVJ due to discomfort or pain during the task.

### Walking gait

Regarding gait velocity and stride length during self-selected gait and standardized gait, there did not appear to be any change in mean gait velocity from pre- to post-intervention; however, stride length appeared to show a small improvement (~2 cm longer stride) from pre- to post-intervention (Table 2).

**Table 2:** Average velocity, stride length, and peak kinematic values for patients with JFM in the first 60 % of stance during standardized gait (*n* = 10) and the land phase of the drop vertical jump (*n* = 8) before and after a neuromuscular training program

	Pre		Post		
	Mean	SD	Mean	SD	*d*
Standardized Gait					
Gait Velocity (m/s)	0.82	0.02	0.83	0.04	0.32
Stride Length (m)	0.85	0.04	0.88	0.04	**0.75**
Hip Flexion (°)	26.74	3.07	27.56	3.46	0.25
Hip Adduction (°)	10.31	0.94	10.29	2.11	−0.01
Hip Internal Rotation (°)	0.15	3.32	−0.01	3.08	−0.05
Knee Flexion (°)	−17.07	5.68	−15.91	6.29	0.19
Knee Abduction (°)	−2.88	2.44	−2.39	2.40	0.20
Knee Internal Rotation (°)	4.38	2.32	4.59	3.15	0.08
Ankle Dorsiflexion (°)	8.50	3.14	7.47	4.03	−0.29
Ankle Eversion (°)	−5.80	1.83	−6.20	1.92	−0.21
Drop Vertical Jump					
Hip Flexion (°)	56.91	10.62	64.90	6.37	**0.91**
Hip Adduction (°)	3.67	3.53	3.17	3.69	−0.14
Hip Internal Rotation (°)	4.96	4.86	4.38	4.29	−0.13
Knee Flexion (°)	−82.10	9.41	−82.32	5.32	−0.03
Knee Abduction (°)	−9.70	5.74	−9.82	5.34	−0.02
Knee Internal Rotation (°)	5.03	7.16	3.83	6.30	−0.18
Ankle Dorsiflexion (°)	30.68	4.06	29.13	4.12	−0.38
Ankle Eversion (°)	1.39	4.09	1.84	2.94	0.13
Trunk Flexion (°)	16.89	6.95	23.62	7.04	**0.96**

*m/s* meters per second; ^o^ degrees; bolded effect sizes denote medium-large effects

No other improvements in walking gait were observable in measured peak kinetic or kinematic variables.

### Knee and Hip strength

Regarding knee and hip strength, participants demonstrated small-moderate improvement with increased peak torques in mean knee extension (small effect, Cohen’s d = 0.22–0.38) and mean hip abduction (moderate effect, Cohen’s d = 0.62–0.63) from pre- to post-treatment (Table 4).

### Functional performance

**Fig. 1:**
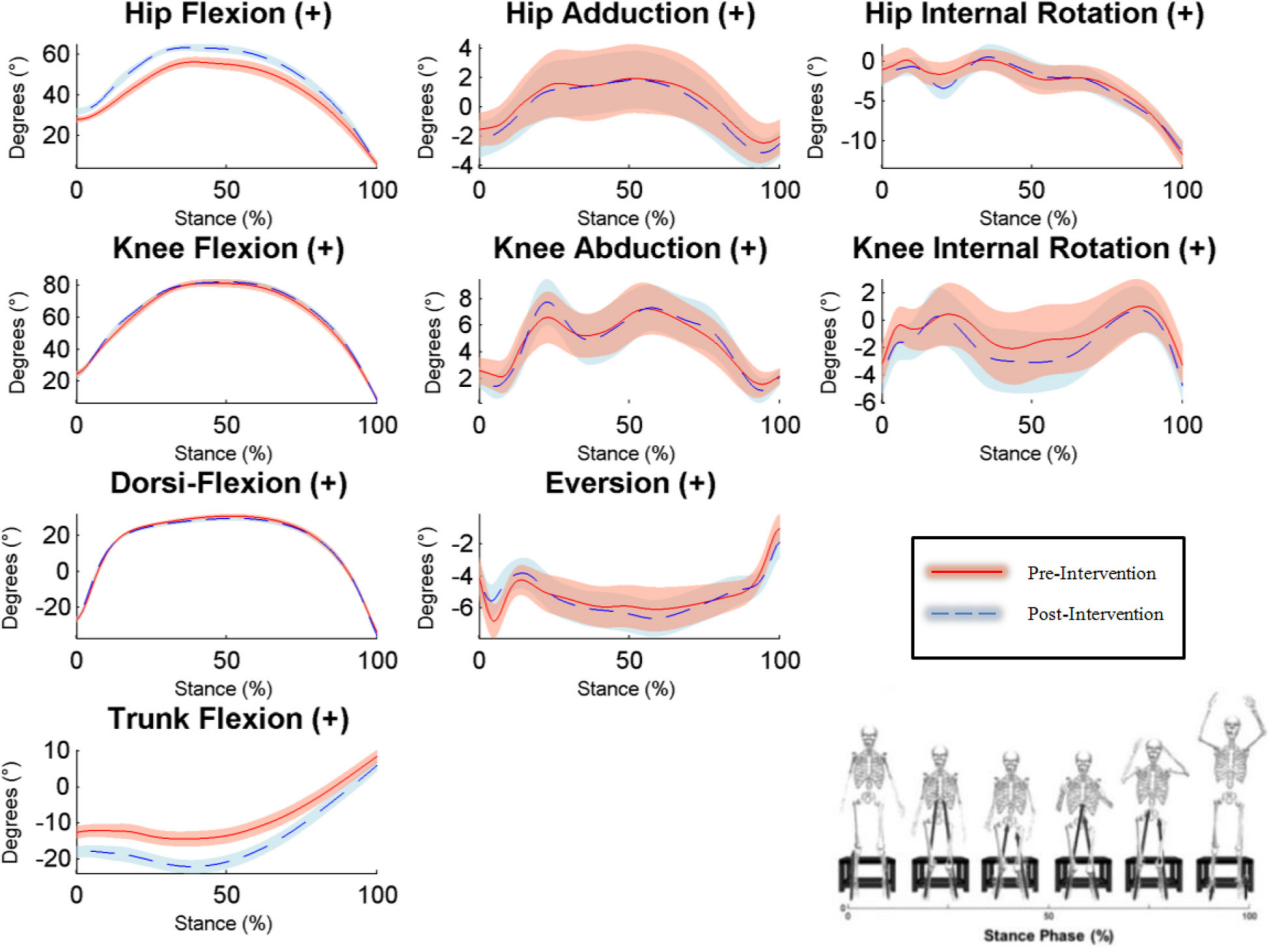
Time series plots of kinematic variables during the stance phase of the DVJ pre- and post-intervention. Graphical representations of the mean performance (represented by solid/broken lines) and (±) SEs (represented by shaded areas) for participants pre- and post-intervention, respectively. Non-overlapping SEs represent significant group differences

**Fig. 2:**
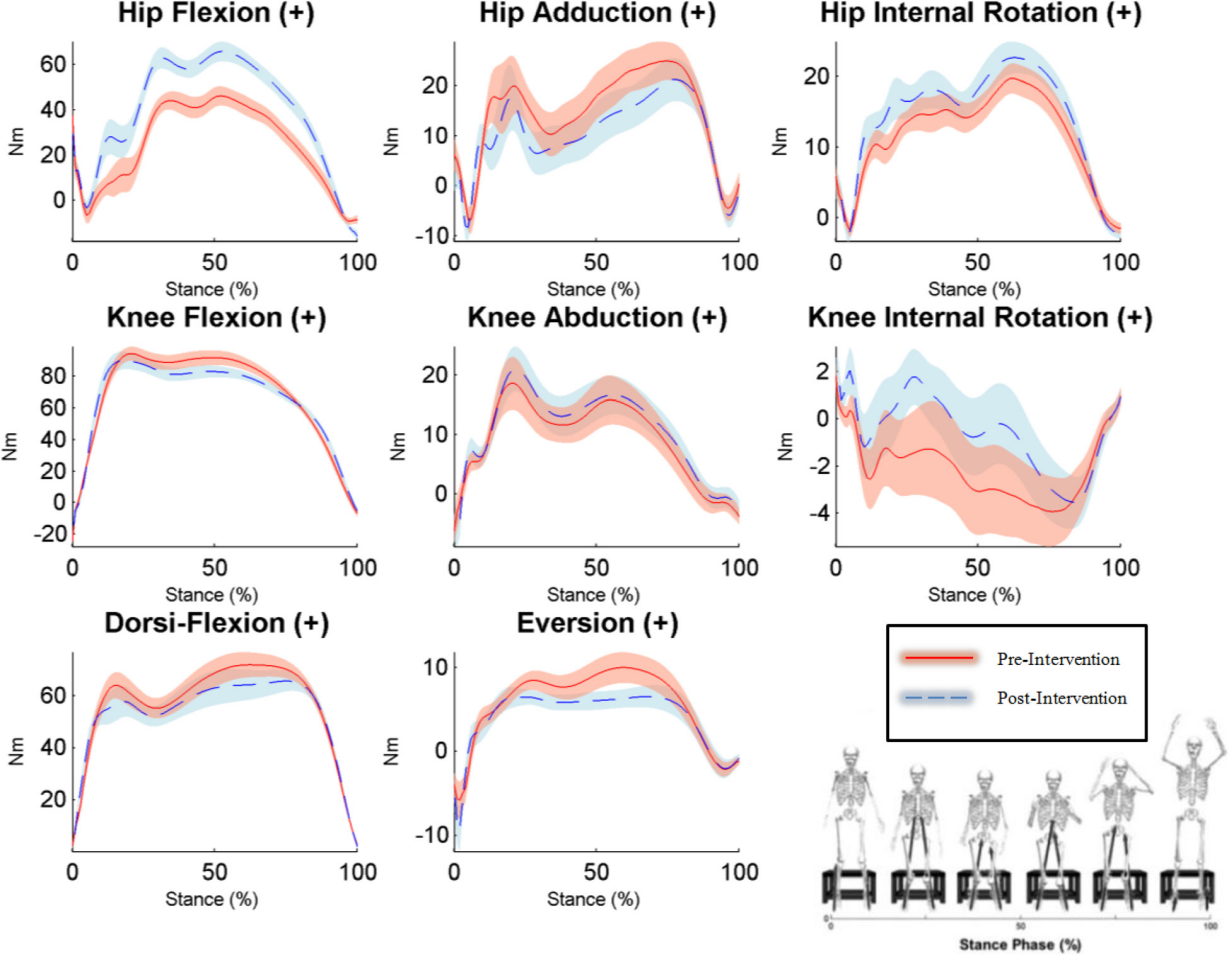
Time series plots of kinetic variables during the stance phase of the DVJ pre- and post-intervention. Graphical representations of the mean performance (represented by solid/broken lines) and (±) SEs (represented by shaded areas) for participants pre- and post-intervention, respectively. Non-overlapping SEs represent significant group differences

Regarding landing technique during the DVJ, maximum hip flexion angle and trunk flexion angle appeared to show strong increases after treatment (Cohen’s d = 0.91 and 0.96 respectively; Table 2 and Fig. 1). Regarding kinetic measures, mean internal hip extensor moment showed a large increase (Cohen’s d = 1.61), while mean ankle eversion showed a moderate decrease Cohen’s d = −0.70); see Table 3 and Fig. 2).

**Table 3:** Average (right and left leg) peak kinetic values for patients with JFM in the first 60 % of stance during standardized gait (*n* = 10) and the land phase of the drop vertical jump (*n* = 8) before and after a neuromuscular training program

	Pre		Post		
	Mean	SD	Mean	SD	*d*
Standardized Gait					
Hip Flexion (Nm)	42.97	10.06	50.84	15.59	**0.60**
Hip Adduction (Nm)	49.84	10.98	58.29	19.76	**0.53**
Hip Internal Rotation (Nm)	5.26	1.72	7.64	3.15	**0.94**
Knee Flexion (Nm)	−24.12	12.61	−26.16	14.35	−0.15
Knee Abduction (Nm)	−3.90	2.23	−4.62	2.31	−0.32
Knee Internal Rotation (Nm)	6.01	2.69	6.44	2.85	0.16
Ankle Dorsiflexion (Nm)	45.21	11.20	48.10	11.24	0.26
Ankle Eversion (Nm)	−7.61	1.90	−8.08	2.53	−0.21
Drop Vertical Jump					
Hip Flexion (Nm)	63.29	7.80	81.51	14.07	**1.61**
Hip Adduction (Nm)	36.66	22.13	32.22	23.04	−0.20
Hip Internal Rotation (Nm)	24.60	6.80	27.65	9.39	0.37
Knee Flexion (Nm)	104.66	17.19	102.90	18.39	−0.10
Knee Abduction (Nm)	25.73	13.27	27.33	11.86	0.13
Knee Internal Rotation (Nm)	5.50	2.94	6.68	2.59	**0.43**
Ankle Dorsiflexion (Nm)	75.80	17.51	73.36	20.93	−0.13
Ankle Eversion (Nm)	12.48	4.36	9.87	2.92	**−0.70**

*Nm* newton-meters; bolded effect sizes denote medium-large effects

### Dynamic postural stability

During the star excursion balance testing, dynamic postural control demonstrated moderate improvement bilaterally, as evidenced by increases in mean composite scores (Cohen’s d = 0.61–0.67; Table 4).

**Table 4:** Star excursion composite scores, isokinetic hip flexion and extension strength measures, and hip abduction strength measures for right and left sides in patients with JFM (*n* = 11) before and after a neuromuscular training program

	Pre		Post		
	Mean	SD	Mean	SD	*d*
Star Excursion (cm)					
Right	81.45	7.24	85.81	7.06	**0.61**
Left	81.02	7.14	85.83	7.19	**0.67**
Knee Extension 300°/s (Nm/kg)					
Right	1.03	0.18	1.10	0.19	0.38
Left	1.01	0.19	1.05	0.18	0.22
Knee Flexion 300°/s (Nm/kg)					
Right	0.71	0.12	0.74	0.15	0.22
Left	0.72	0.13	0.73	0.18	0.06
Hip Abduction (Nm/kg)					
Right	0.73	0.28	0.90	0.26	**0.63**
Left	0.72	0.23	0.91	0.37	**0.62**

*cm* centimeters, *Nm/kg* newton-meters per kilogram; bolded effect sizes denote medium-large effects

## Discussion

The results of this study provide initial evidence for the utility of objective biomechanical assessments in measuring the effects of an 8-week integrated neuromuscular and CBT intervention on a range of measures of gait mechanics, knee and hip strength, functional performance and biomechanics, and dynamic postural stability in youth with JFM. Notably, participants demonstrated medium to large effect sizes in improved hip flexion and internal rotation gait mechanics, improved hip strength, improved hip and trunk flexion and ankle eversion during a functional landing task, and increased dynamic postural stability from pre- to post-treatment. These results not only demonstrate the promise of improved biomechanics in youth with JFM after the FIT Teens program, but this study also moves the field forward by utilizing a sophisticated assessment battery to examine improvements in movement mechanics in JFM. Few studies have examined biomechanics in fibromyalgia, and those that do tend to focus on one or two areas of muscle strength or control [47–49] rather than integrating multiple aspects of performance. In the context of the larger literature, the findings of the present study offer promise for improvement across multiple domains, and the sophisticated biomechanical assessments used in this study offer the opportunity to examine small changes which may translate into greater physical functioning.

Objective improvement in biomechanical performance may allow for safer and more stable execution of functional tasks of daily living, such as sit-to-standing, walking, stair climbing, etc., while minimizing risk of overexertion, stress, injury, and/or pain in individuals with chronic pain. The exercises in the FIT Teens program were specifically chosen to properly engage muscle groups that are essential to performing functional movements [43]. For instance, during the DVJ participants showed increases in hip flexion position and external hip flexion moment post-treatment, suggesting proper activation of the posterior chain and therefore, a safer landing mechanism (e.g., more effective deceleration and improved function during the landing phase of a dynamic movement). Likewise, the increased net hip internal rotator moment during walking gait signified enhanced recruitment of the hip external rotators, primarily the gluteus maximus and piriformis, which optimizes lower body alignment in such a way that maintains the knee position in line with the hips, as opposed to a valgus or “knock-knee” position which may leave a patient at higher risk of injury [50] and the development of anterior knee pain [51, 52]. Consequently, participants may also be able perform common activities of daily living that recruit the posterior chain and hip external rotators with improved mechanics and lower risk of injury, such as ascending/descending stairs. Notably, adolescents with JFM and comorbid joint hypermobility were able to safely engage in the neuromuscular exercise training using modifications that ensured neutral joint positioning to protect against hyperextension [44]. The findings of improved joint strength and joint positioning may translate into the ability to safely engage in physical exercise and prevent joint injury for such patients.

Results of this study can also be interpreted with respect to known areas of biomechanical deficits in youth with JFM compared to their healthy peers. In a prior study, compared to healthy peers, adolescents with JFM were found to have significant deficits in knee and hip strength (particularly in hip abduction) and functional performance [22]. These specific areas appeared to show improvement after the FIT Teens intervention, particularly improved hip abduction strength and improved functional performance. Regarding hip abduction strength, there was a medium effect size for JFM participants improving hip strength, bringing them closer to healthy control subjects [22] by the end of the intervention. Improved trunk flexion during the DVJ post-treatment was also noted to look more similar to healthy control landing mechanics. These results suggest that through the course of the FIT Teens intervention, adolescents with JFM were able to progress towards normalized strength and biomechanics, which may translate into safer and more comfortable functional movement.

Information gathered from this study may be used to better design intervention protocols to target specific aspects of biomechanical functioning, as well as to inform health care providers and families about what aspects of biomechanics may be more difficult to correct for teens with JFM. Considering that the goal of this intervention was to improve integrated functional movement, by beginning with basic fundamental movement skills and confidence in movement, it is promising that postural stability and landing mechanics demonstrated improvement. Conversely, it is not surprising that relatively few of the biomechanical measures of walking gait demonstrated improvement given that there was less impairment in the walking gait of adolescents with JFM relative to healthy peers [22]. When considered in contrast to more complex functional tasks, interventionists may be able to focus less on walking gait (which may not require as much attention) in order to target more challenging movements, which showed improvement after the FIT Teens intervention.

### Limitations

Although the results of this pilot study suggest that assessment measures were capable of detecting biomechanical changes after intervention, the sample size was small and there was no control group in this feasibility study. Further replication is needed to test whether improvements were statistically significant and could be more definitively attributed to the treatment. Another limitation of the current study is the brief nature of the intervention—eight weeks may not be a long enough intervention to establish lasting changes in functional biomechanics. Still, there were signals that biomechanical changes were occurring in desirable direction for improved physical functioning.

### Future directions

Future studies should examine how these objective changes in biomechanical performance may translate into improved daily functioning. It is unknown how much improvement in biomechanics is needed to enable one to participate in more physical exercise and activities of daily living. For example, use of actigraphy (using an accelerometer to assess time spent in vigorous and moderate activity) is one way of objectively monitoring physical activity to determine whether biomechanical changes correspond to actual increases in physical activity. Future studies would also benefit from use of a control group and, in order to determine whether changes after treatment are maintained over time, follow-up assessments should be conducted to assess biomechanics and physical functioning. Also, retention might be improved by offering sessions later in the afternoon/evening to prevent scheduling conflicts. In our current work, we are also including more formal screening for suicidal ideation which may suggest the need for more intensive mental health services to stabilize emotional status prior to participation. Finally, future studies may utilize larger sample sizes in order to examine how these objective measures of biomechanics and physical functioning relate to subjective reports of functional disability and confidence in movement.

## Conclusions

Overall, results of this study offer initial evidence for the utility of biomechanical assessment measures in a complex pediatric chronic pain population. This sophisticated approach allowed for objective assessment of observable changes in biomechanical performance after an 8-week integrated CBT and neuromuscular training intervention for youth with JFM. Sophisticated measurement of biomechanics may offer enhanced capabilities to better design intervention protocols specifically targeting biomechanical deficits in patients with musculoskeletal pain for optimal outcomes. Results suggest that through the FIT Teens intervention, adolescents with JFM can potentially progress towards normalized strength and biomechanics, which may translate into improved movement confidence and engagement with physical exercise.

## Abbreviations

CBT, cognitive behavioral therapy; cm, centimeter; DVJ, drop vertical jump task; FIT Teens, fibromyalgia integrative training for teens; JFM, juvenile fibromyalgia; Kg, kilogram; M, mean; Nm, newton-meter; SD, standard deviation

## Additional file

Additional file 1: Balance and Strength database. (XLSX 16 kb)
